# Miscellaneous

**Published:** 1890-06

**Authors:** 


### MISCELLANEOUS.

### WHY THE LEAVES CHANGE COLOR.

"Probably not one person in a thousand knows why leaves change their color in the fall," remarked an eminent botanist the other day. "The common and old-fashioned indea is that all this red and golden glory we see now is caused by frosts. A true and scientific explanation of the causes of the coloring of leaves would necessitate a long and intricate discussion. Stated briefly and in proper language, the causes are these: The green matter in the tissue of a leaf is composed of two colors, red and blue. When the sap ceases to flow in the autumn, and the natural growth of the trees ceases, oxidation of the tissues take place. Under certain conditions the green of the leaf changes to red, under different conditions it takes on a yellow or brown tint. The difference in color is due to the difference in combination of the original constituents of the green tissue, and to the varying conditions of climate, exposure and soil. A dry, cold climate produces more brilliant foliage than one that is damp and warm. This is the reason that American autumns are so much more gorgeous than those of Scotland and England. There are several things about leaves than even science cannot explain. For instance, why one of two trees growing side by side, of the same age and having the same exposure, should take on a brilliant red in the fall, and the other should turn yellow; or why one branch of a tree should be highly colored and the rest of the tree have only a yellow tint, are questions that are as impossible to answer, as why one member of a family should be perfectly healthy and another sickly. Maples and oaks have brightest colors."

### PAPER MAKING AN OLD ART.

By the research of Dr. Julius Weissner, paper making is proved to have originated in the East. By a careful microscopical examination of ancient Oriental parchments, he has discovered that linen rags were used for this purpose as early as the eighth and ninth centuries. The process of "claying," too, which has been thought a modern device among paper makers, obtained at that period.

---

Adam Clark, in returning thanks at the table of another, made use of the following significant and pertinent words: "Lord bless these vegetables and this fruit and bread; and if thou canst bless under the gospel what thou didst curse under the law, bless this swine's flesh also." If people would follow the example of this eminent man, there would be less sour stomachs, and sour faces, and sour experiences generally.

A Safe Investment.—E. C. Morris and Co's Fire and Burglar Proof Safes, advertised in our journal, are acknowledged to be of the best. Since this Co. began to advertise with us they have been compelled to enlarge their means of supply. The Salem fire was a great card for this safe.

### PREACHING IN A TRANCE.

The statement has been extensively published that Major Perry, an illiterate and ignorant Edgefield county, South Carolina negro, while in what appears to be a trance, preaches learned and eloquent sermons. He is attracting a great deal of attention, and several enterprising citizens of Edgefield county have the human phenomenon in charge, and are exhibiting him to large audiences. Perry goes to bed, and after a few moments of apparently sound slumber his muscles begin to twitch, his limbs to contract, and his body becomes distorted in unseemly shapes. This spasm soon passes off, and then he begins to preach. He takes his text from the Bible, naming book, chapter and verse, all the time lying flat on his back, with his eyes shut, and for half an hour or more preaches, using strictly grammatical and even eloquent language. At the conclusion of his sermon he sings a hymn to an old air, the words being new. Then comes a prayer and dismissal of the congregation in the usual manner of religious bodies.

---

### AGAINST CAPITAL PUNISHMENT.

A petition is in circulation in England for the abolition of capital punishment. The reasons set forth by the petitioners are these :—

1st. Because capital punishment does not prevent or diminish the crime of murder.

2nd. Because such a form of death is a relic of barbarism, and completely out of touch with the national opinion and feeling of this nineteenth century.

3rd. Because the error of cutting short the life of innocent persons (too often occurring) is an irreparable wrong to the nation.

4th. Because violent death by strangulation (even if it satisfies the vengeance of imperfect human law) is a national disgrace, and is opposed to the highest instincts of humanity.

---

THE "DIVINING-ROD."—Professor E. Ray Lankester, having recently expressed some doubts upon the alleged powers of a boy "water-finder" who has been in the employ of the Grinton Mining Company, in the North of England, the chairman of the company, Dr. M'Clure, has replied to them, denying emphatically that the boy, whose name is Rodwell, is an imposter. He says that the lad when tested never fails to find either water or mineral veins, the lodes having always been found exactly at the places indicated. The "divining-rod" which he holds moves only in obedience to the muscular contractions of his hands, and a rod of any kind of wood, or even of any material substance whatever, can be used, provided it be a conductor of electricity. Rodwell usually walks with his hands tightly clasped before him, and as soon as he steps upon a mineral vein or water, he is powerless to unclasp them until he moves away from the region of the lode of conduit. The lad is about fourteen years of age. These statements by Dr. M'Clure have excited considerable comment in Yorkshire.

SIX MILLION DEAD LETTERS.—There were more than 6,000,000 letters received at the Dead Letter Office last year. Five hundred thousand were never called for at the post offices to which they were addressed, 150,000 were sent in by hotel keepers because their departing guests failed to leave their new addresses, 120,000 were insufficiently prepaid for mileage, 400,000 were erroneously or illegibly addressed, while 17,000 bore no subscriptions whatever. Eighteen thousand contained money amounting to $35,000 in all, and 22,000 contained drafts, checks, etc., amounting to $1,600,000.

### THE ALPINE ROSE.

From the German of Teodor Löwe.

Translated for HALL'S JOURNAL OF HEALTH.

Beneath the mountain's snowy brow,
'Mid mosses sear and ices rude,
The Alpine rose is blooming now,
A poem sweet of solitude.

The spring airs may not thither roam,
Its young leaves kissing and beguiling;
As one that's lost it stands alone
Upon the rampart sweetly smiling.

In the eternal silence there,
Far upward gleam the glacier walls,
By tempest builded layer on layer,
To guard its chill prismatic halls.

O blest is he whom fortune spares,
Amid life's evanescent doom,
At peace above all earthly cares,
Like this sweet flower to live and bloom.

